# Antacid exposure and immunotherapy outcomes among patients with advanced hepatocellular carcinoma

**DOI:** 10.1177/17588359211010937

**Published:** 2021-04-28

**Authors:** Tomi Jun, Umut Ozbek, Sirish Dharmapuri, Camille Hardy-Abeloos, Huili Zhu, Jung-Yi Lin, Nicola Personeni, Tiziana Pressiani, Naoshi Nishida, Pei-Chang Lee, Chieh-Ju Lee, Hannah Hildebrand, Neil Nimkar, Sonal Paul, Petros Fessas, Muntaha Naeem, Dominik Bettinger, Uqba Khan, Anwaar Saeed, Yi-Hsiang Huang, Masatoshi Kudo, Lorenza Rimassa, Thomas U. Marron, David J. Pinato, Celina Ang

**Affiliations:** Division of Hematology and Medical Oncology, Tisch Cancer Institute, Icahn School of Medicine at Mount Sinai, New York, NY, USA; Department of Population Health Science and Policy, Icahn School of Medicine at Mount Sinai, New York, NY, USA; Division of Hematology and Medical Oncology, Tisch Cancer Institute, Icahn School of Medicine at Mount Sinai, New York, NY, USA; Icahn School of Medicine at Mount Sinai, New York, NY, USA; Department of Medicine, Icahn School of Medicine at Mount Sinai, New York, NY, USA; Department of Population Health Science and Policy, Icahn School of Medicine at Mount Sinai, New York, NY, USA; Department of Biomedical Sciences, Humanitas University, Pieve Emanuele, Milan, Italy; Medical Oncology and Hematology Unit, Humanitas Cancer Center, IRCCS Humanitas Research Hospital, Rozzano, Milan, Italy; Medical Oncology and Hematology Unit, Humanitas Cancer Center, IRCCS Humanitas Research Hospital, Rozzano, Milan, Italy; Department of Gastroenterology and Hepatology, Kindai University Faculty of Medicine, Osaka-Sayama, Osaka, Japan; Division of Gastroenterology and Hepatology, Department of Medicine, Taipei; Veterans General Hospital, Institute of Clinical Medicine, School of Medicine, National Yang-Ming University, Taipei; Division of Gastroenterology and Hepatology, Department of Medicine, Taipei; Veterans General Hospital, Institute of Clinical Medicine, School of Medicine, National Yang-Ming University, Taipei; Division of Medical Oncology, Department of Medicine, Kansas University Cancer Center, Westwood, KS, USA; New York Presbyterian Brooklyn Methodist Hospital, Brooklyn, NY, USA; New York Presbyterian Brooklyn Methodist Hospital, Brooklyn, NY, USA; Department of Surgery and Cancer, Imperial College London, Hammersmith Hospital, London, UK; Department of Surgery and Cancer, Imperial College London, Hammersmith Hospital, London, UK; Department of Medicine II, Faculty of Medicine, Medical Center University of Freiburg, University of Freiburg, Freiburg, Germany; Division of Hematology and Oncology, Weill Cornell Medicine/New York Presbyterian Hospital, New York, NY, USA; Division of Medical Oncology, Department of Medicine, Kansas University Cancer Center, Westwood, KS, USA; Division of Gastroenterology and Hepatology, Department of Medicine, Taipei; Veterans General Hospital, Institute of Clinical Medicine, School of Medicine, National Yang-Ming University, Taipei; Department of Gastroenterology and Hepatology, Kindai University Faculty of Medicine, Osaka-Sayama, Osaka, Japan; Department of Biomedical Sciences, Humanitas University, Pieve Emanuele, Milan, Italy; Medical Oncology and Hematology Unit, Humanitas Cancer Center, IRCCS Humanitas Research Hospital, Rozzano, Milan, Italy; Division of Hematology and Medical Oncology, Tisch Cancer Institute, Icahn School of Medicine at Mount Sinai, New York, NY, USA; Imperial Centre for Translational and Experimental Medicine (ICTEM), 72 Du Cane Road, White City, London, W12 0NN, UK Division of Oncology, Department of Translational Medicine, Piemonte Orientale University, Novara, Italy; Division of Hematology and Medical Oncology, Tisch Cancer Institute, Icahn School of Medicine at Mount Sinai, 1 Gustave Levy Place, Box 1079, New York, NY 10029, USA

**Keywords:** antacid, hepatocellular carcinoma, immunotherapy, proton pump inhibitor

## Abstract

**Background:**

Antibiotic exposure has been associated with worse outcomes with immune checkpoint inhibitors (ICIs) in cancer patients, likely due to disruption of the gut microbiome. Other commonly prescribed medications, such as proton pump inhibitors (PPIs) and histamine-2-receptor antagonists (H2RAs), are also known to disrupt the microbiome, but data on their association with ICI outcomes are conflicting.

**Methods:**

We conducted a retrospective, multicenter, international cohort study including 314 hepatocellular carcinoma (HCC) patients treated with ICIs from 2017 to 2019 to assess the association between PPI or H2RA exposure (up to 30 days before ICI) and overall survival. Secondary outcomes included overall response rate (ORR) and development of any treatment-related adverse events (AEs).

**Results:**

Baseline PPI/H2RA exposure was not associated with overall survival in univariable (HR 1.01, 95% CI 0.75–1.35) or multivariable analysis (HR 0.98, 95% CI 0.71–1.36). Baseline PPI/H2RA exposure was not associated with either ORR (OR 1.32, 95% CI 0.66–2.65) or AEs (OR 1.07, 95% CI 0.54–2.12) in multivariable analysis.

**Conclusions:**

Our results suggest that exposure to PPI/H2RA prior to ICIs does not adversely affect outcomes in HCC patients.

## Introduction

There is growing interest in potential interactions between commonly prescribed medications, such as antibiotics, proton pump inhibitors (PPIs), steroids, and opioids, and cancer immunotherapy.^1^ Antibiotic exposure has been repeatedly linked to worse immunotherapy outcomes.^2–8^ Experimental evidence suggests that this association is mediated through the gut microbiome, which is disrupted by antibiotics.^5,9–12^ Several other commonly prescribed medication classes, including antacids such as PPIs and histamine-2-receptor antagonists (H2RAs), are also known to alter the gut microbiome,^13–16^ and recent reports have linked PPI usage to worse immunotherapy outcomes in lung and bladder cancer.^7,17,18^

Immune checkpoint inhibitors (ICIs) have become an important component of hepatocellular carcinoma (HCC) management. ICI agents were initially approved by the US Food and Drug Administration in 2017 for second-line treatment of advanced HCC on the basis of promising overall response rates (ORRs) of 20% in early phase studies.^19,20^ More recently, the combination of atezolizumab and bevacizumab became the first immunotherapy regimen to improve overall survival (OS) over sorafenib in advanced HCC in a phase III trial and has become standard of care.^21^ However, ORRs remain limited, underlining the need to identify markers and mediators of ICI resistance and response.

Given the increasing use of ICIs in HCC management and the lack of published data addressing the effect of antacids on outcomes in this setting, we conducted this observational study to test the associations of PPI/H2RA exposure before ICI treatment with survival and response.

## Methods

### Study population

The study population comprised HCC patients treated with immunotherapy between 2017 and 2019 at 11 tertiary referral centers in the United States, Europe, and Asia. An earlier version of the cohort with fewer sites was described previously.^22^ Included patients had a diagnosis of HCC in accordance with American Association for the study of Liver Disease^23^ and European Association for the Study of the Liver^24^ guidelines, received systemic ICI therapy (either monotherapy or in combination), and had measurable disease according to RECIST 1.1^25^ criteria at ICI commencement. Baseline antacid data were not available from two US sites, both of which were excluded (*N* = 102). An additional eight patients were excluded due to missing baseline antacid data.

Clinical variables were obtained through manual review of electronic medical records by investigators at each site using a standardized data collection form, including specific fields for concomitant medications such as antacids. Antacids were defined as PPIs (omeprazole, lansoprazole, dexlansoprazole, esomeprazole, pantoprazole, rabeprazole) or H2RAs (famotidine, ranitidine, cimetidine, nizatidine). Data were censored on 20 February 2020. Disease staging was conducted by computerized tomography or magnetic resonance imaging prior to ICI initiation and at approximately 9-week intervals during treatment.

This study was conducted in accordance with the Declaration of Helsinki. The Institutional Review Board (IRB) at Imperial College London acted as the central IRB, whose review was accepted by all participating institutions’ IRBs (Ref. 17/WA/0161/R18009) (Supplemental Table 1). The central IRB determined that this research involved no greater than minimal risk and approved a waiver for informed consent.

### Study design

This was a retrospective cohort study with a primary outcome of OS, measured from the date of initiation of ICI to the date of death from any cause or last follow-up. Secondary outcomes were ORR, defined as the proportion of patients with a best response of either complete response (CR) or partial response (PR) by RECIST v1.1 criteria; disease control rate (DCR), defined as CR, PR, or stable disease (SD) by RECIST v1.1 criteria; and the development of treatment-related adverse events (AEs) of any grade. AEs were identified from clinical notes in conjunction with laboratory and radiographic evidence. Events were deemed treatment-related based on known side-effect profiles of ICI drugs and the judgment of the treating physician, with study investigators validating the association during chart review. AEs were graded following the National Cancer Institute Common Terminology Criteria for Adverse Events *versus* 5.0. All outcome data were obtained from the electronic medical records of the individual institutions.

The primary predictor in the analysis was baseline antacid (H2RA or PPI) exposure within 30 days prior to ICI initiation. Exposure was defined as an active prescription in the medical record per clinical notes or prescription records. Data were also collected on antacid exposures concurrent with ICI treatment; that is, antacid exposures between the dates of ICI initiation and ICI cessation. Baseline H2RA and PPI exposure were also considered separately as secondary predictors. Additional clinically relevant covariates were age, sex, alpha-fetoprotein (AFP) >400 ng/ml, presence of cirrhosis (clinically or radiologically diagnosed), Barcelona Clinic Liver Cancer (BCLC) stage (A–B *versus* C–D), Child–Turcotte Pugh class, Eastern Cooperative Oncology Group (ECOG) performance status (0 *versus* ⩾1), and antibiotic exposure within 30 days prior to ICI.

### Statistical analysis

Descriptive statistics were summarized using medians and interquartile ranges (IQRs) for continuous variables, and frequency and proportions for categorical variables. The Wilcoxon rank-sum test and Fisher’s exact test were used to compare the distribution of continuous and categorical variables between antacid exposures, respectively. AFP values were missing in 17 patients and were imputed as the median value. Median OS was determined using the Kaplan–Meier method, and Kaplan–Meier survival curves were compared using the log-rank test.

Univariable and multivariable Cox proportional hazards models were used to test the association of the predictors with OS. Some covariates for the multivariable model were selected *a priori*, based on the number of events in the dataset and existing literature. These covariates were: age, sex, geographic region, AFP > 400 ng/ml, BCLC (A–B *versus* C–D), and prior antibiotics, in addition to the primary predictor. Additional covariates significant in the univariable regression analysis were added to the multivariable model, provided they were not redundant with covariates already being included, for example, BCLC stage incorporates Child–Pugh score and performance status. The proportional hazards assumption was tested and the final model was stratified by geographic region; that is, separate baseline hazard functions were estimated for each geographic region. There was no evidence of multicollinearity with variance inflation factors of all predictors <5. A summary of each model was presented using hazard ratios (HRs) and 95% confidence intervals (CIs). Subgroup analyses were conducted using a model including the interaction term between antacid exposure and the grouping variable. The HR and 95% CI for antacid exposure in each subgroup was presented, along with the *p* value of the interaction term.

Univariable and multivariable logistic regression models were used to test the association of predictors with the secondary outcomes ORR, DCR, and AE. A similar set of covariates were selected for the multivariable models *a priori*, with additional covariates added based on significance in univariable analysis, absence of collinearity, and available degrees of freedom. A summary of each model was presented using odds ratios (OR) and 95% CIs.

All analyses were conducted in R version 4.0.0 (Vienna, Austria).

## Results

### Baseline characteristics

The cohort included 314 patients with HCC treated with ICIs between 2017 and 2019 at centers in Asia (*N* = 99, 31.5%), the United States (*N* = 154, 49%), and Europe (*N* = 61, 19.4%). Clinical characteristics of the cohort are reported in Table 1.

**Table 1.** Baseline characteristics, by baseline antacid exposure.

Variable	No antacid exposure (*N* = 204)	Antacid exposure (*N* = 110)	*p* value	All patients (*N* = 314)
Age (years)	65.6 (59.1–72.6)	67.9 (58.2–70.7)	0.77	66 (58.7–71.6)
Male	161 (78.9%)	87 (79.1%)	1	248 (79%)
USA	119 (58.3%)	35 (31.8%)	<0.001	154 (49%)
Europe	23 (11.3%)	38 (34.5%)	<0.001	61 (19.4%)
Asia	62 (30.4%)	37 (33.6%)	0.634	99 (31.5%)
PD-1 monotherapy	186 (91.2%)	85 (77.3%)	<0.001	271 (86.3%)
PD-1/CTLA-4 Combination	8 (3.9%)	13 (11.8%)	0.016	21 (6.7%)
PD-1/TKI Combination	10 (4.9%)	12 (10.9%)	0.063	22 (7%)
First-line ICI	93 (45.6%)	44 (40%)	0.404	137 (43.6%)
Second-line ICI	102 (50%)	56 (50.9%)	0.906	158 (50.3%)
Third-line or later	9 (4.4%)	10 (9.1%)	0.135	19 (6.1%)
Prior sorafenib	110 (53.9%)	65 (59.1%)	0.406	175 (55.7%)
Prior local therapy	182 (89.2%)	95 (86.4%)	0.467	277 (88.2%)
Cirrhosis	147 (72.1%)	78 (70.9%)	0.896	225 (71.7%)
Chronic hepatitis B	57 (27.9%)	31 (28.2%)	1	88 (28%)
Chronic hepatitis C	76 (37.3%)	42 (38.2%)	0.903	118 (37.6%)
HBV/HCV co-infection	6 (2.9%)	2 (1.8%)	0.718	8 (2.5%)
Non-viral liver disease	65 (31.9%)	35 (31.8%)	1	100 (31.8%)
Alcoholic liver disease	35 (17.2%)	23 (20.9%)	0.447	58 (18.5%)
Non-alcoholic steatohepatitis	21 (10.3%)	9 (8.2%)	0.688	30 (9.6%)
Other liver disease	7 (3.4%)	4 (3.6%)	1	11 (3.5%)
ECOG ⩾ 1	88 (43.1%)	57 (51.8%)	0.155	145 (46.2%)
BCLC A	5 (2.5%)	0 (0%)	0.167	5 (1.6%)
BCLC B	51 (25%)	30 (27.3%)	0.686	81 (25.8%)
BCLC C	146 (71.6%)	77 (70%)	0.795	223 (71%)
BCLC D	2 (1%)	3 (2.7%)	0.348	5 (1.6%)
Child–Pugh A	137 (67.2%)	78 (70.9%)	0.441	215 (68.5%)
Child–Pugh B	58 (28.4%)	27 (24.5%)	0.507	85 (27.1%)
Child–Pugh C	7 (3.4%)	3 (2.7%)	1	10 (3.2%)
Portal venous thrombosis	68 (33.3%)	40 (36.4%)	0.535	108 (34.4%)
Extrahepatic metastasis	103 (50.5%)	59 (53.6%)	0.637	162 (51.6%)
No measurable intrahepatic disease	26 (12.7%)	9 (8.2%)	0.261	35 (11.1%)
Multifocal (⩾3) intrahepatic nodules	115 (56.4%)	59 (53.6%)	0.632	174 (55.4%)
Maximum diameter of largest lesion (cm)	6.15 (3–12.5)	6 (3.6–10.1)	0.663	6 (3.2–11.2)
Alpha-fetoprotein (ng/ml)	183.3 (10.9–2563.5)	223.65 (14.25–4615.5)	0.408	183.3 (12.4–2917.7)
Baseline PPI only^†^	NA	85 (78%)	NA	85 (27.1%)
Baseline H2RA only	NA	17 (15.6%)	NA	17 (5.4%)
Baseline PPI and H2RA exposure	NA	7 (6.4%)	NA	7 (2.2%)
No concurrent antacid^‡^	147 (72.4%)	10 (9.3%)	<0.001	157 (50.5%)
Concurrent PPI only	34 (16.7%)	73 (67.6%)	<0.001	107 (34.4%)
Concurrent H2RA only	9 (4.4%)	19 (17.6%)	<0.001	28 (9%)
Concurrent PPI and H2RA exposure	13 (6.4%)	6 (5.6%)	1	19 (6.1%)
Baseline antibiotic exposure	14 (6.9%)	26 (23.6%)	<0.001	40 (12.7%)
Baseline steroid exposure	4 (2%)	10 (9.1%)	0.006	14 (4.5%)

Data are presented as medians and interquartile ranges or counts and proportions.

† One patient had baseline antacid exposure, but the type of antacid was not documented.

‡ Two patients with baseline antacid exposure had missing data on concurrent antacid exposure; one patient without baseline antacid exposure had missing data on concurrent antacid exposure.

BCLC, Barcelona Clinic Liver Cancer; CTLA-4, cytotoxic T-lymphocyte-associated protein 4; ECOG, Eastern Cooperative Oncology Group; H2RA, histamine-2-receptor antagonist; HBV, hepatitis B virus; HCV, hepatitis C virus; ICI, immune checkpoint inhibitor; PD-1, programmed cell death protein 1; PPI, proton pump inhibitor; TKI, tyrosine kinase inhibitor.

The cohort was predominantly male (*N* = 248, 79%), with a median age of 66 years (IQR 59–72). At baseline, 225 (71.7%) patients had clinical or radiographic evidence of cirrhosis. The most common underlying causes of liver disease were hepatitis C virus (*N* = 118, 37.6%) and hepatitis B virus (*N* = 88, 28%), followed by alcoholic liver disease (*N* = 58, 18.5%) and non-alcoholic steatohepatitis (*N* = 30, 9.6%). Liver function was preserved (Child–Pugh A) in most patients (*N* = 215, 68.5%).

Most patients (*N* = 223, 71%) met criteria for BCLC stage C HCC at the time of ICI initiation. The majority of patients were treated with anti-PD-1 monotherapy (*N* = 271, 86.3%). Half of the patients (*N* = 158, 50.3%) had received one prior systemic treatment, whereas 43.6% (*N* = 137) were naïve to systemic treatment. Most patients (*N* = 277, 88.2%) had also had prior local therapy, most commonly surgical resection (*N* = 103, 32.8%).

### Treatment outcomes

There were 190 deaths (60.5%) during a median follow-up of 9.2 months (IQR 4.0–16.1). Based on best radiographic response, there were 123 patients with SD (39.2%), 31 with PRs (9.9%), and 21 with CRs (6.7%), resulting in a DCR of 55.7% and an ORR of 16.6%. Median OS in the whole cohort was 12.3 months (95% CI 9.9–15.7). The median duration of ICI treatment was 3.7 months (IQR 1.9–8.6). The most common reason for treatment discontinuation was progressive disease (*N* = 178, 56.7%). Treatment-related AEs developed in 33.8% of patients (*N* = 106), with 15.6% of patients (*N* = 49) experiencing AEs of grade 2 or higher. The most common AEs involved the liver (*N* = 41, 13.1%) and skin (*N* = 33, *N* = 10.5%).

### Antacid exposures

Baseline exposure to antacids, either a PPI or a H2RA, within 30 days prior to ICI treatment was present in 35% of patients (*N* = 110) (Table 1). Most baseline exposures were to PPIs (*N* = 85, 27.1%), but some were exposed to H2RAs (*N* = 17, 5.4%), or both (*N* = 7, 2.2%). Among those with baseline antacid exposure, 90.9% (*N* = 100) also had antacid exposure concurrent with their ICI treatment.

Additional details on duration and indications for antacid usage were available for 224 patients, 76 of whom had baseline antacid exposure and 117 of whom had concurrent antacid exposure (Supplemental Tables 2 and 3). Most baseline antacids were prescribed for more than 4 weeks (77.6%), with the most common indications being acid reflux or dyspepsia (43.4%) and procedures or other prophylactic reasons (48.7%). Most concurrent antacid exposures originated from prescriptions beginning before ICI treatment (63.6%) and the most common indications were also acid reflux or dyspepsia (37.9%) and procedures or other prophylaxis (37.1%).

There were baseline differences between the patients with or without antacid exposures (Table 1). Antacid-exposed patients were more commonly from European sites (34.5% of exposed *versus* 11.3% of unexposed, *p* < 0.001) and less commonly from US sites (31.8% of exposed *versus* 58.3% of unexposed, *p* < 0.001). Antacid-exposed patients were less likely to have been treated with anti-PD-1 monotherapy (77.3% of exposed *versus* 91.2% of unexposed, *p* < 0.001), but more likely to have been treated with a combination of anti-PD-1 and anti-CTLA-4 agents (11.8% of exposed, 3.9% of unexposed, *p* = 0.02). Antacid-exposed patients were also more likely to have been exposed to antibiotics (23.6% of exposed *versus* 6.9% of unexposed, *p* < 0.001) or steroids (9.1% of exposed *versus* 2.0% of unexposed, *p* = 0.006) in the 30 days prior to ICI initiation.

### Association of baseline antacid use with immunotherapy outcomes

In univariable analysis, baseline antacid exposure was not associated with OS (HR 1.01, 95% CI 0.75–1.35), nor was baseline PPI exposure (HR 1.14, 95% CI 0.84–1.54) or H2RA exposure (0.58, 95% CI 0.31–1.10) (Table 2). Factors associated with OS were: ECOG PS > 0 (HR 1.55, 95% CI 1.15–2.09), BCLC C or D (HR 1.45, 95% CI 1.04–2.03), Child–Pugh class B or C (HR 1.94, 95% CI 1.43–2.62), portal venous thrombosis (HR 1.73, 95% CI 1.29–2.32), multifocal (⩾3) intrahepatic nodules (HR 1.69, 95% CI 1.26–2.26), and AFP > 400 ng/ml (HR 1.39, 95% CI 1.04–1.85).

**Table 2.** Univariable and multivariable Cox proportional hazards models for overall survival.

Variable	Univariable HR (95% CI)	*p* value	Multivariable HR^*^ (95% CI)	*p* value
Age (years)	0.997 (0.99–1.01)	0.642	1.00 (0.99–1.01)	0.958
Male	1.07 (0.75–1.54)	0.701	1.12 (0.77–1.61)	0.560
Geographic region				
Europe *versus* USA	0.97 (0.67–1.41)	0.866	−	−
Asia *versus* USA	0.83 (0.60–1.17)	0.292	−	−
Immunotherapy treatment				
PD-1/CTLA-4 *versus* PD-1	0.78 (0.44–1.37)	0.380	−	−
PD-1/TKI *versus* PD-1	0.80 (0.45–1.41)	0.443	−	−
Second-line or later	1.15 (0.86–1.54)	0.339	−	−
Cirrhosis	1.06 (0.77–1.45)	0.740	−	−
Liver disease				
HCV *versus* HBV	0.85 (0.60–1.22)	0.388	−	−
HBV/HCV *versus* HBV	0.16 (0.02–1.19)	0.074	−	−
Non-viral *versus* HBV	1.09 (0.76–1.56)	0.650	−	−
ECOG ⩾ 1	1.55 (1.15–2.09)	0.004	−	−
BCLC C/D	1.45 (1.04–2.03)	0.030	1.22 (0.85–1.75)	0.276
Child–Pugh B/C	1.94 (1.43–2.62)	<0.001	−	−
Portal venous thrombosis	1.73 (1.29–2.32)	<0.001	1.51 (1.10–2.08)	0.011
Extrahepatic metastasis	1.06 (0.80–1.42)	0.673	−	−
⩾3 intrahepatic nodules	1.69 (1.26–2.26)	<0.001	1.83 (1.34–2.50)	<0.001
Maximum diameter of largest lesion (cm)	0.998 (0.99–1.00)	0.503	−	−
Alpha-fetoprotein >400 ng/ml	1.39 (1.04–1.85)	0.025	1.31 (0.96–1.79)	0.084
Baseline antacid exposure	1.01 (0.75–1.35)	0.971	0.98 (0.71–1.36)	0.909
Baseline PPI exposure	1.14 (0.84–1.54)	0.409	−	−
Baseline H2RA exposure	0.58 (0.31–1.10)	0.095	−	−
Baseline antibiotic exposure	1.38 (0.92–2.06)	0.122	1.23 (0.78–1.94)	0.370

* Stratified by geographic region.

BCLC, Barcelona Clinic Liver Cancer; CI, confidence interval; CTLA-4, cytotoxic T-lymphocyte-associated protein 4; ECOG, Eastern Cooperative Oncology Group; H2RA, histamine-2-receptor antagonist; HBV, hepatitis B virus; HCV, hepatitis C virus; HR, hazard ratio; PD-1, programmed cell death protein 1; PPI, proton pump inhibitor; TKI, tyrosine kinase inhibitor.

In multivariable analysis, baseline antacid exposure remained unassociated with OS (HR 0.98, 95% CI 0.71–1.36) (Table 2). Significant independent predictors of survival in this model were multifocal intrahepatic disease (HR 1.83, 95% CI 1.34–2.50) and portal venous thrombosis (HR 1.51, 95% CI 1.10–2.08).

**Figure 1.**
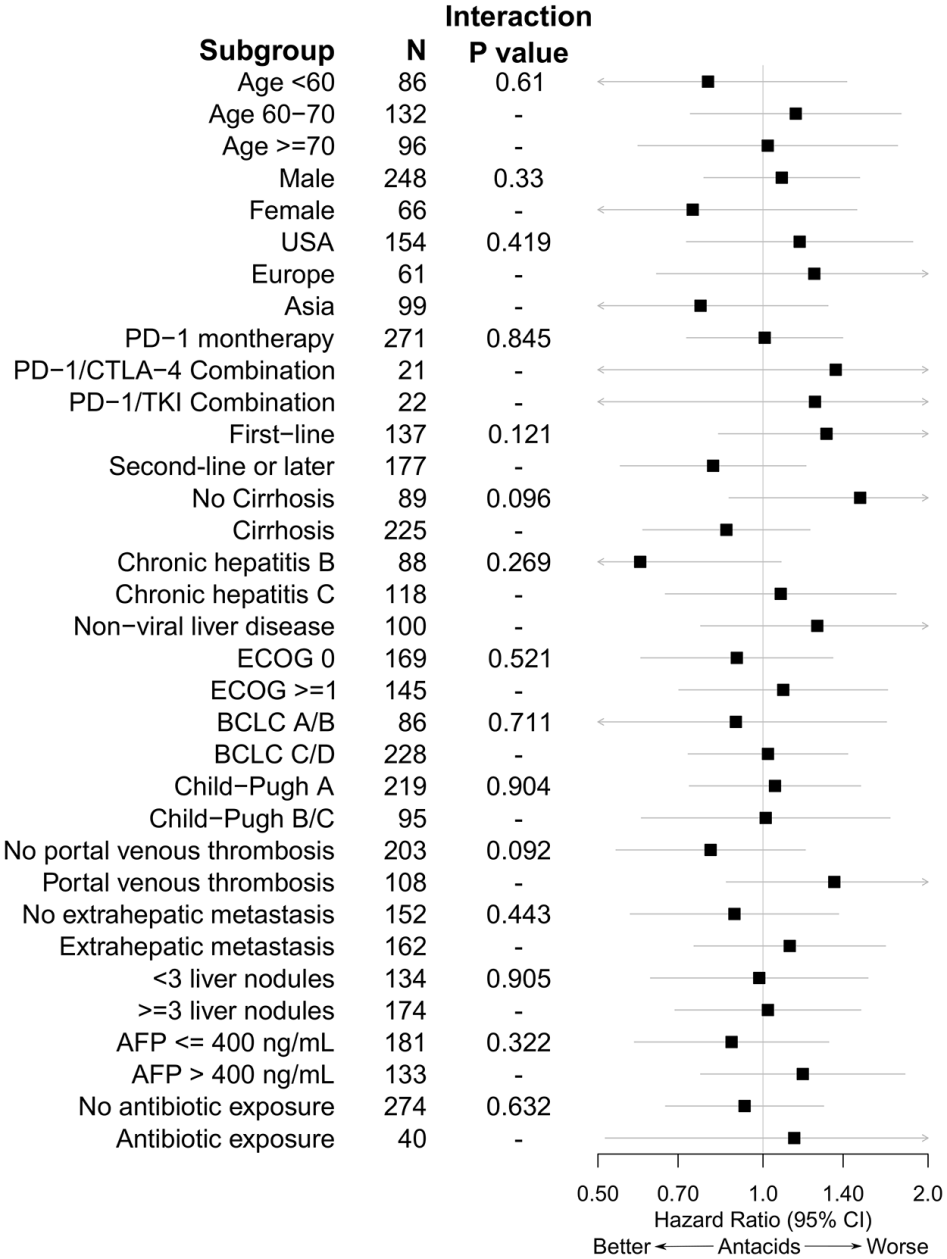
Cox proportional hazards models for overall survival taking into account interactions between baseline antacid exposure and subgroup membership. Hazard ratios and 95% confidence intervals for antacid exposure are presented along with *p* values for the interaction term in each mod.

In subgroup analyses, there were trends towards heterogeneous effects of antacid exposure based on cirrhosis status (interaction *p* = 0.096) and the presence or absence of portal venous thrombosis (interaction *p* = 0.092) (Figure 1).

To control more strictly for possible confounding by baseline antibiotic exposure, we repeated the OS analysis while excluding the 40 patients who had received baseline antibiotics. Baseline antacid use was not associated with OS in univariable analysis (HR 0.92, 95% CI 0.66–1.29) nor multivariable analysis (HR 0.92, 95% CI 0.63–1.34) after excluding antibiotic-exposed patients.

Baseline antacid exposure was not associated with either ORR (univariable OR 1.06, 95% CI 0.57–1.98; multivariable OR 1.32, 95% CI 0.66–2.65) or DCR (univariable OR 1.56, 95% CI 0.95–2.57; multivariable OR 1.34, 95% CI 0.75–2.39). There were no significant associations with ORR in either univariable or multivariable analysis (Table 3). DCR was associated with AFP > 400 ng/ml (OR 0.57, 95% CI 0.36–0.9), European sites (OR 3.97, 95% CI 1.91–8.24), and chronic hepatitis C (OR 2.04, 95% CI 1.14–3.66) in univariable analysis. AFP > 400 ng/ml (OR 0.55, 95% CI 0.33–0.92) and European sites (OR 3.94, 95% CI 1.73–9.03) remained independently associated with DCR in multivariable analysis (Table 4).

**Table 3.** Univariable and multivariable logistic regression for overall response.

Variable	Univariable OR (95% CI)	*p* value	Multivariable OR (95% CI)	*p* value
Age (years)	0.99 (0.97–1.02)	0.459	0.99 (0.97–1.02)	0.534
Male	0.95 (0.45–1.97)	0.883	0.88 (0.41–1.85)	0.730
Geographic region				
Europe *versus* USA	0.63 (0.26–1.53)	0.306	0.53 (0.20–1.44)	0.213
Asia *versus* USA	1.28 (0.66–2.48)	0.462	1.16 (0.58–2.35)	0.676
Immunotherapy treatment				
PD-1/CTLA-4 *versus* PD-1	1.90 (0.70–5.17)	0.209	−	−
PD-1/TKI *versus* PD-1	0.53 (0.12–2.36)	0.403	−	−
Second line or later	0.83 (0.45–1.51)	0.537	−	−
Cirrhosis	1.74 (0.83–3.67)	0.142	−	−
Liver disease				
HCV *versus* HBV	0.94 (0.46–1.9)	0.852	−	−
HBV/HCV *versus* HBV	0.77 (0.08–6.99)	0.812	−	−
Non-viral *versus* HBV	0.56 (0.25–1.25)	0.159	−	−
ECOG ⩾ 1	1.08 (0.59–1.97)	0.799	−	−
BCLC C/D	0.83 (0.43–1.59)	0.567	−	−
Child–Pugh B/C	1.33 (0.70–2.51)	0.383	−	−
Portal venous thrombosis	1.04 (0.55–1.95)	0.905	−	−
Extrahepatic metastasis	0.85 (0.47–1.55)	0.602	−	−
⩾3 intrahepatic nodules	0.66 (0.36–1.22)	0.185	−	−
Maximum diameter of largest lesion (cm)	1.00 (0.99–1.01)	0.994	−	−
Alpha-fetoprotein >400 ng/ml	0.95 (0.51–1.74)	0.858	−	−
Baseline antacid exposure	1.06 (0.57–1.98)	0.847	1.32 (0.66–2.65)	0.429
Baseline PPI exposure	1.15 (0.60–2.18)	0.676	−	−
Baseline H2RA exposure	1.10 (0.36–3.42)	0.865	−	−
Baseline antibiotic exposure	0.67 (0.25–1.82)	0.435	0.60 (0.20–1.74)	0.343

BCLC, Barcelona Clinic Liver Cancer; CI, confidence interval; CTLA-4, cytotoxic T-lymphocyte-associated protein 4; ECOG, Eastern Cooperative Oncology Group; H2RA, histamine-2-receptor antagonist; HBV, hepatitis B virus; HCV, hepatitis C virus; OR, odds ratio; PD-1, programmed cell death protein 1; PPI, proton pump inhibitor; TKI, tyrosine kinase inhibitor.

**Table 4.** Univariable and multivariable logistic regression for disease control rate.

Variable	Univariable OR (95% CI)	*p* value	Multivariable OR (95% CI)	*p* value
Age (years)	1.02 (1.00–1.05)	0.028	1.01 (0.99–1.03)	0.472
Male	0.98 (0.55–1.74)	0.930	1.16 (0.62–2.18)	0.648
Geographic region				
Europe *versus* USA	3.97 (1.91–8.24)	<0.001	3.94 (1.73–9.03)	0.001
Asia *versus* USA	1.2 (0.70–2.04)	0.509	1.40 (0.75–2.60)	0.296
Immunotherapy treatment				
PD-1/CTLA-4 *versus* PD-1	2.02 (0.76–5.37)	0.160	−	−
PD-1/TKI *versus* PD-1^*^	−	−	−	−
Second line or later	1.08 (0.68–1.73)	0.741	−	−
Cirrhosis	1.02 (0.60–1.71)	0.955	−	−
Liver disease				
HCV *versus* HBV	2.04 (1.14–3.66)	0.016	1.88 (0.95–3.71)	0.069
HBV/HCV *versus* HBV	5.26 (0.59–46.9)	0.138	8.25 (0.84–81.9)	0.070
Non-viral *versus* HBV	1.55 (0.85–2.81)	0.152	1.08 (0.54–2.16)	0.836
ECOG ⩾ 1	0.89 (0.56–1.43)	0.637	−	−
BCLC C/D	0.88 (0.52–1.49)	0.635	0.93 (0.52–1.63)	0.790
Child–Pugh B/C	0.78 (0.47–1.29)	0.325	−	−
Portal venous thrombosis	0.94 (0.57–1.54)	0.802	−	−
Extrahepatic metastasis	1.07 (0.67–1.7)	0.790	−	−
⩾3 Intrahepatic nodules	0.66 (0.41–1.06)	0.083	−	−
Maximum diameter of largest lesion (cm)	1.01 (0.997–1.02)	0.198	−	−
Alpha-fetoprotein >400 ng/ml	0.57 (0.36–0.92)	0.021	0.55 (0.33–0.92)	0.024
Baseline antacid exposure	1.56 (0.95–2.57)	0.079	1.34 (0.75–2.39)	0.332
Baseline PPI exposure	1.31 (0.79–2.2)	0.299	−	−
Baseline H2RA exposure	2.29 (0.82–6.44)	0.115	−	−
Baseline antibiotic exposure	0.72 (0.37–1.44)	0.355	0.63 (0.29–1.39)	0.252

* All 20 patients treated with PD-1/TKI attained disease control.

BCLC, Barcelona Clinic Liver Cancer; CI, confidence interval; CTLA-4, cytotoxic T-lymphocyte-associated protein 4; ECOG, Eastern Cooperative Oncology Group; H2RA, histamine-2-receptor antagonist; HBV, hepatitis B virus; HCV, hepatitis C virus; OR, odds ratio; PD-1, programmed cell death protein 1; PPI, proton pump inhibitor; TKI, tyrosine kinase inhibitor.

Baseline antacid exposure was associated with development of any AE (OR 1.85, 95% CI 1.14–3.05) in univariable analysis, but not multivariable analysis (OR 1.07, 95% CI 0.54–2.12) (Table 5). Predictors independently associated with development of any AE were European site (OR 8.33, 95% CI 2.64–26.4) and maximum diameter of the largest nodule in centimeters (OR 1.05, 95% CI 1.03–1.07). Compared with anti-PD-1 monotherapy, combination treatments were associated with AEs in univariable (PD-1/CTLA-4 OR 5.1, 95% CI 1.98–13.1; PD-1/TKI 8.17, 95% CI 2.89–23.1) but not multivariable analysis (PD-1/CTLA-4 OR 1.08, 95% CI 0.29–4.06; PD-1/TKI OR 1.68, 95% CI 0.41–6.89).

**Table 5.** Univariable and multivariable logistic regression for any treatment-related adverse event.

Variable	Univariable OR (95% CI)	*p* value	Multivariable OR (95% CI)	*p* value
Age (years)	1.02 (0.995–1.04)	0.131	0.999 (0.97–1.03)	0.960
Male	0.66 (0.38–1.16)	0.149	0.69 (0.33–1.47)	0.341
Geographic region				
Europe *versus* USA	9.58 (4.84–18.9)	<0.001	8.33 (2.64–26.4)	<0.001
Asia *versus* USA	2.14 (1.21–3.81)	0.009	0.61 (0.24–1.55)	0.295
Immunotherapy treatment				
PD-1/CTLA-4 *versus* PD-1	5.10 (1.98–13.1)	<0.001	1.08 (0.29–4.06)	0.908
PD-1/TKI *versus* PD-1	8.17 (2.89–23.1)	<0.001	1.68 (0.41–6.89)	0.469
Second line or later	0.96 (0.60–1.53)	0.848	−	−
Cirrhosis	0.75 (0.45–1.26)	0.277	−	−
Liver disease				
HCV *versus* HBV	1.90 (1.02–3.51)	0.042	1.69 (0.74–3.86)	0.213
HBV/HCV *versus* HBV^*^	−	−	−	−
Non-viral *versus* HBV	2.30 (1.22–4.32)	0.010	1.37 (0.58–3.25)	0.475
ECOG ⩾ 1	0.55 (0.34–0.89)	0.014	−	−
BCLC C/D	0.88 (0.52–1.48)	0.634	0.70 (0.37–1.35)	0.288
Child–Pugh B/C	0.65 (0.38–1.09)	0.104	−	−
Portal venous thrombosis	0.92 (0.56–1.51)	0.732	−	−
Extrahepatic metastasis	1.23 (0.77–1.97)	0.384	−	−
⩾3 intrahepatic nodules	0.81 (0.51–1.30)	0.386	−	−
Maximum diameter of largest lesion (cm)	1.03 (1.01–1.04)	<0.001	1.05 (1.03–1.07)	<0.001
Alpha-fetoprotein >400 ng/ml	1.40 (0.87–2.25)	0.161	1.15 (0.62–2.13)	0.650
Baseline antacid exposure	1.85 (1.14–3.01)	0.013	1.07 (0.54–2.12)	0.850
Baseline PPI exposure	1.84 (1.11–3.05)	0.018	−	−
Baseline H2RA exposure	0.63 (0.24–1.63)	0.338	−	−
Baseline antibiotic exposure	0.71 (0.34–1.48)	0.356	1.17 (0.44–3.1)	0.750

* No patients with HBV/HCV co-infection (*N* = 8) developed treatment-related adverse events.

BCLC, Barcelona Clinic Liver Cancer; CI, confidence interval; CTLA-4, cytotoxic T-lymphocyte-associated protein 4; ECOG, Eastern Cooperative Oncology Group; H2RA, histamine-2-receptor antagonist; HBV, hepatitis B virus; HCV, hepatitis C virus; OR, odds ratio; PD-1, programmed cell death protein 1; PPI, proton pump inhibitor; TKI, tyrosine kinase inhibitor.

## Discussion

In this multicenter retrospective cohort study of patients with advanced HCC and treated with ICI therapy, there was no association between OS and exposure to PPIs or H2RAs within 30 days prior to ICI initiation. Furthermore, there were no significant associations between antacid exposure and secondary outcomes such as OR, disease control, or AEs.

Previous observational studies of antacid exposure and immunotherapy outcomes have yielded mixed results. Several single-center retrospective studies focusing on patients with non-small cell lung cancer (NSCLC) or melanoma have failed to find an association between PPI use and immunotherapy outcome.^26–29^ Two of these studies were able to detect significant associations between immunotherapy outcome and antibiotic use, but not PPI exposure.^27,29^ Another study using a combination of retrospective and early phase clinical trial data found significant associations between outcomes and antibiotic exposure, but not PPI exposure.^5^

Conversely, *post hoc* analyses of phase II and III clinical trial data have found independent associations between PPI exposure and immunotherapy outcomes. Using data from the Checkmate 069 trial comparing ipilimumab *versus* ipilimumab with nivolumab in melanoma, Homicsko *et al*.^30^ found significantly reduced ORR, progression-free survival, and OS among PPI-exposed patients; these associations were maintained in multivariable analysis. Chalabi *et al*.^7^ and Hopkins *et al*.^17^ have also recently reported large analyses showing negative associations between PPI exposure and atezolizumab outcomes in lung and bladder cancer, respectively.

Chalabi *et al*.^7^ used data from the OAK and POPLAR trials of atezolizumab *versus* chemotherapy to examine the impact of antibiotic and PPI exposure in 1512 immunotherapy-treated patients with NSCLC. They found that both antibiotic and PPI exposure were independently associated with reduced OS among atezolizumab-treated patients, but not chemotherapy-treated patients. However, the interaction between PPIs and treatment was not significant.

Hopkins *et al*.^17^ examined PPI use as a predictor of outcomes among 1360 bladder cancer patients treated with atezolizumab or chemotherapy in the IMvigor210 and IMvigor211 trials. They similarly found that PPI exposure was an independent predictor of worse OS and PFS in atezolizumab-treated but not chemotherapy-treated patients; in this case, the interaction between PPI and treatment was statistically significant. Overall, these analyses from clinical trials are the most compelling evidence thus far that PPI usage can influence immunotherapy outcome in solid tumors.

Unlike prior studies, our study focuses on patients with HCC. The question of antacid exposure and immunotherapy is particularly relevant in HCC for both clinical and mechanistic reasons. Patients with chronic liver disease and HCC are often prescribed PPIs, whether they may be clinically indicated or not.^31^ Our results suggest that those with indications for PPIs may use them prior to immunotherapy without adversely affecting outcomes.

Mechanistically, the negative findings of our study raise questions of whether the immune microenvironment or microbiome in HCC differs from lung and bladder cancer to the extent that medication exposures have less influence on immunotherapy outcomes. The liver has been recognized as a site of myeloid-derived suppressor cell accumulation,^32^ and liver metastases have been associated with worse immunotherapy outcomes in lung cancer.^33,34^ Both cirrhosis and HCC are associated with altered gut microbiota and the microbiome has been linked with progression of cirrhosis to HCC.^35–38^

It is possible that the baseline dysbiosis of cirrhotic HCC patients limits the impact of medication-induced perturbations to the microbiome. Intriguingly, we found a suggestive but non-significant interaction (*p* = 0.096) between antacid exposure and cirrhosis status with regards to OS. One recent study of eight HCC patients reported microbial differences between immunotherapy-responders and non-responders but excluded patients with advanced liver disease.^39^ More microbiome data from immunotherapy-treated patients are needed to understand the interactions between medication exposures, the microbiome, and immunotherapy outcomes in HCC.

Another mechanistic link between PPIs and immunotherapy outcomes may be through the pH of the tumor microenvironment. Pre-clinical studies have linked the acidity of the tumor microenvironment with T-cell anergy and immune escape.^40,41^ Experiments in mice have demonstrated that systemic PPIs can increase intra-tumoral pH,^42^ which can in turn improve response to immunotherapy.^41^ The net effect of PPIs on immunotherapy outcomes may depend on the balance between detrimental impacts on the microbiome *versus* beneficial changes in the tumor microenvironment.

The external validity of these findings is bolstered by the size and geographic diversity of this cohort. Unlike clinical trials, which have been mostly limited to patients with preserved liver function, this observational cohort reflects a real-world clinical population. In-depth characterization of each patient’s tumor also allowed us to control for potential confounders such as performance status, liver function, disease stage, and antibiotic exposure.

This study has several limitations. Although our cohort size compares favorably with other retrospective studies examining this question,^26–29^ power may have been limited to detect significant effects. Our definition of antacid exposure did not incorporate duration or dose of antacid treatment, and medication adherence could not be assessed from the medical record alone. However, this definition and its limitations is consistent the definition of exposure used in prior studies, including the large studies of Chalabi *et al*.^7^ and Hopkins *et al*.^17^ Although we captured antibiotic and steroid use, we did not record use of other common medications which may also influence the microbiome or the immune system. Ultimately, this retrospective analysis was exploratory and the findings are hypothesis-generating. Untangling causal relationships between medications, the microbiome, and cancer immunotherapy will require larger retrospective clinical datasets, in-depth mechanistic studies, and randomized clinical trials, where feasible.

In conclusion, these observational data suggest that PPIs and H2RAs do not adversely impact ICI therapy outcomes in patients with advanced HCC. These findings require validation in prospective cohorts and provide motivation for mechanistic studies to dissect the interactions of medication exposures and the microbiome in HCC as compared to other solid tumors.

## Supplemental Material

sj-docx-1-tam-10.1177_17588359211010937 – Supplemental material for Antacid exposure and immunotherapy outcomes among patients with advanced hepatocellular carcinoma Click here for additional data file. Supplemental material, sj-docx-1-tam-10.1177_17588359211010937 for Antacid exposure and immunotherapy outcomes among patients with advanced hepatocellular carcinoma by Tomi Jun, Umut Ozbek, Sirish Dharmapuri, Camille Hardy-Abeloos, Huili Zhu, Jung-Yi Lin, Nicola Personeni, Tiziana Pressiani, Naoshi Nishida, Pei-Chang Lee, Chieh-Ju Lee, Hannah Hildebrand, Neil Nimkar, Sonal Paul, Petros Fessas, Muntaha Naeem, Dominik Bettinger, Uqba Khan, Anwaar Saeed, Yi-Hsiang Huang, Masatoshi Kudo, Lorenza Rimassa, Thomas U. Marron, David J. Pinato and Celina Ang in Therapeutic Advances in Medical Oncology

sj-docx-2-tam-10.1177_17588359211010937 – Supplemental material for Antacid exposure and immunotherapy outcomes among patients with advanced hepatocellular carcinoma Click here for additional data file. Supplemental material, sj-docx-2-tam-10.1177_17588359211010937 for Antacid exposure and immunotherapy outcomes among patients with advanced hepatocellular carcinoma by Tomi Jun, Umut Ozbek, Sirish Dharmapuri, Camille Hardy-Abeloos, Huili Zhu, Jung-Yi Lin, Nicola Personeni, Tiziana Pressiani, Naoshi Nishida, Pei-Chang Lee, Chieh-Ju Lee, Hannah Hildebrand, Neil Nimkar, Sonal Paul, Petros Fessas, Muntaha Naeem, Dominik Bettinger, Uqba Khan, Anwaar Saeed, Yi-Hsiang Huang, Masatoshi Kudo, Lorenza Rimassa, Thomas U. Marron, David J. Pinato and Celina Ang in Therapeutic Advances in Medical Oncology

sj-docx-3-tam-10.1177_17588359211010937 – Supplemental material for Antacid exposure and immunotherapy outcomes among patients with advanced hepatocellular carcinoma Click here for additional data file. Supplemental material, sj-docx-3-tam-10.1177_17588359211010937 for Antacid exposure and immunotherapy outcomes among patients with advanced hepatocellular carcinoma by Tomi Jun, Umut Ozbek, Sirish Dharmapuri, Camille Hardy-Abeloos, Huili Zhu, Jung-Yi Lin, Nicola Personeni, Tiziana Pressiani, Naoshi Nishida, Pei-Chang Lee, Chieh-Ju Lee, Hannah Hildebrand, Neil Nimkar, Sonal Paul, Petros Fessas, Muntaha Naeem, Dominik Bettinger, Uqba Khan, Anwaar Saeed, Yi-Hsiang Huang, Masatoshi Kudo, Lorenza Rimassa, Thomas U. Marron, David J. Pinato and Celina Ang in Therapeutic Advances in Medical Oncology

## References

[1] Hussain N Naeem M Pinato DJ. Concomitant medications and immune checkpoint inhibitor therapy for cancer: causation or association? Hum Vaccin Immunother. Epub ahead of print 23 June 2020. DOI: 10.1080/21645515.2020.1769398. PMC7872020 32574106

[2] Tinsley N Zhou C Tan G, et al. Cumulative antibiotic use significantly decreases efficacy of checkpoint inhibitors in patients with advanced cancer. Oncologist. Epub ahead of print 10 July 2019. DOI: 10.1634/theoncologist.2019-0160. PMC6964118 31292268

[3] Pinato DJ Howlett S Ottaviani D, et al. Association of prior antibiotic treatment with survival and response to immune checkpoint inhibitor therapy in patients with cancer. JAMA Oncol 2019; 5: 1774–1778. 31513236 10.1001/jamaoncol.2019.2785 PMC6743060

[4] Greally M Chou JF Chatila WK, et al. Clinical and molecular predictors of response to immune checkpoint inhibitors in patients with advanced esophagogastric cancer. Clin Cancer Res 2019; 25: 6160–6169. 31337644 10.1158/1078-0432.CCR-18-3603 PMC6905384

[5] Routy B Le Chatelier E Derosa L, et al. Gut microbiome influences efficacy of PD-1-based immunotherapy against epithelial tumors. Science 2018; 359: 91–97. 29097494 10.1126/science.aan3706

[6] Hopkins AM Kichenadasse G Karapetis CS, et al. Concomitant antibiotic use and survival in urothelial carcinoma treated with atezolizumab. Eur Urol 2020; 78: 540–543. 32660748 10.1016/j.eururo.2020.06.061

[7] Chalabi M Cardona A Nagarkar DR, et al. Efficacy of chemotherapy and atezolizumab in patients with non-small-cell lung cancer receiving antibiotics and proton pump inhibitors: pooled post hoc analyses of the OAK and POPLAR trials. Ann Oncol 2020; 31: 525–531. 32115349 10.1016/j.annonc.2020.01.006

[8] Derosa L Hellmann MD Spaziano M, et al. Negative association of antibiotics on clinical activity of immune checkpoint inhibitors in patients with advanced renal cell and non-small-cell lung cancer. Ann Oncol 2018; 29: 1437–1444. 29617710 10.1093/annonc/mdy103 PMC6354674

[9] Vétizou M Pitt JM Daillère R, et al. Anticancer immunotherapy by CTLA-4 blockade relies on the gut microbiota. Science 2015; 350: 1079–1084. 26541610 10.1126/science.aad1329 PMC4721659

[10] Gopalakrishnan V Spencer CN Nezi L, et al. Gut microbiome modulates response to anti-PD-1 immunotherapy in melanoma patients. Science 2018; 359: 97–103. 29097493 10.1126/science.aan4236 PMC5827966

[11] Matson V Fessler J Bao R, et al. The commensal microbiome is associated with anti-PD-1 efficacy in metastatic melanoma patients. Science 2018; 359: 104–108. 29302014 10.1126/science.aao3290 PMC6707353

[12] Sivan A Corrales L Hubert N, et al. Commensal Bifidobacterium promotes antitumor immunity and facilitates anti–PD-L1 efficacy. Science 2015; 350: 1084–1089. 26541606 10.1126/science.aac4255 PMC4873287

[13] Jackson MA Goodrich JK Maxan M-E, et al. Proton pump inhibitors alter the composition of the gut microbiota. Gut 2016; 65: 749–756. 26719299 10.1136/gutjnl-2015-310861 PMC4853574

[14] Imhann F Bonder MJ Vich Vila A, et al. Proton pump inhibitors affect the gut microbiome. Gut 2016; 65: 740–748. 26657899 10.1136/gutjnl-2015-310376 PMC4853569

[15] Dial S Delaney JAC Barkun AN, et al. Use of gastric acid-suppressive agents and the risk of community-acquired Clostridium difficile-associated disease. JAMA 2005; 294: 2989–2995. 16414946 10.1001/jama.294.23.2989

[16] Gupta RW Tran L Norori J, et al. Histamine-2 receptor blockers alter the fecal microbiota in premature infants. J Pediatr Gastroenterol Nutr 2013; 56: 397–400. 23254444 10.1097/MPG.0b013e318282a8c2

[17] Hopkins AM Kichenadasse G Karapetis CS, et al. Concomitant proton pump inhibitor use and survival in urothelial carcinoma treated with atezolizumab. Clin Cancer Res 2020; 26: 5487–5493. 32933995 10.1158/1078-0432.CCR-20-1876

[18] Buti S Bersanelli M Perrone F, et al. Effect of concomitant medications with immune-modulatory properties on the outcomes of patients with advanced cancer treated with immune checkpoint inhibitors: development and validation of a novel prognostic index. Eur J Cancer 2021; 142: 18–28. 33212418 10.1016/j.ejca.2020.09.033

[19] Zhu AX Finn RS Edeline J, et al. Pembrolizumab in patients with advanced hepatocellular carcinoma previously treated with sorafenib (KEYNOTE-224): a non-randomised, open-label phase 2 trial. Lancet Oncol 2018; 19: 940–952. 29875066 10.1016/S1470-2045(18)30351-6

[20] El-Khoueiry AB Sangro B Yau T, et al. Nivolumab in patients with advanced hepatocellular carcinoma (CheckMate 040): an open-label, non-comparative, phase 1/2 dose escalation and expansion trial. Lancet 2017; 389: 2492–2502. 28434648 10.1016/S0140-6736(17)31046-2 PMC7539326

[21] Finn RS Qin S Ikeda M, et al. Atezolizumab plus bevacizumab in unresectable hepatocellular carcinoma. N Engl J Med 2020; 382: 1894–1905. 32402160 10.1056/NEJMoa1915745

[22] Pinato DJ Kaneko T Saeed A, et al. Immunotherapy in hepatocellular cancer patients with mild to severe liver dysfunction: adjunctive role of the ALBI Grade. Cancers (Basel) 2020; 12: 1862. 10.3390/cancers12071862 PMC7408648 32664319

[23] Heimbach JK Kulik LM Finn RS, et al. AASLD guidelines for the treatment of hepatocellular carcinoma. Hepatology 2018; 67: 358–380. 28130846 10.1002/hep.29086

[24] European Association for the Study of the Liver. EASL clinical practice guidelines: management of hepatocellular carcinoma. J Hepatol 2018; 69: 182–236. 29628281 10.1016/j.jhep.2018.03.019

[25] Eisenhauer EA Therasse P Bogaerts J, et al. New response evaluation criteria in solid tumours: revised RECIST guideline (version 1.1). Eur J Cancer 2009; 45: 228–247. 19097774 10.1016/j.ejca.2008.10.026

[26] Mukherjee S Ibrahimi S Khalid B, et al. Do proton pump inhibitors modulate the efficacy of anti-PD-1/PD-L1 therapy? A retrospective study. J Oncol Pharm Pract 2019; 25: 762–764. 29690815 10.1177/1078155218771152

[27] Hakozaki T Okuma Y Omori M, et al. Impact of prior antibiotic use on the efficacy of nivolumab for non-small cell lung cancer. Oncol Lett 2019; 17: 2946–2952. 30854072 10.3892/ol.2019.9899 PMC6365976

[28] Trabolsi A Winter M Rodriguez E. Proton pump inhibitors and response to immune check-point inhibitors: single center study. JCO 2019; 37 (Suppl. 15): e14092.

[29] Zhao S Gao G Li W, et al. Antibiotics are associated with attenuated efficacy of anti-PD-1/PD-L1 therapies in Chinese patients with advanced non-small cell lung cancer. Lung Cancer 2019; 130: 10–17. 30885328 10.1016/j.lungcan.2019.01.017

[30] Homicsko K Richtig G Tuchmann F, et al. LBA2 Proton pump inhibitors negatively impact survival of PD-1 inhibitor based therapies in metastatic melanoma patients. Ann Oncol 2018; 29(Suppl. 10): 1.

[31] Cole HL Pennycook S Hayes PC. The impact of proton pump inhibitor therapy on patients with liver disease. Aliment Pharmacol Ther 2016; 44: 1213–1223. 27774677 10.1111/apt.13827

[32] Ilkovitch D Lopez DM. The liver is a site for tumor induced myeloid-derived suppressor cell accumulation and immunosuppression. Cancer Res 2009; 69: 5514–5521. 19549903 10.1158/0008-5472.CAN-08-4625 PMC2706931

[33] Cortellini A Tiseo M Banna GL, et al. Clinicopathologic correlates of first-line pembrolizumab effectiveness in patients with advanced NSCLC and a PD-L1 expression of ⩾50%. Cancer Immunol Immunother 2020; 69: 2209–2221. 32474768 10.1007/s00262-020-02613-9 PMC11027629

[34] Botticelli A Cirillo A Scagnoli S, et al. The agnostic role of site of metastasis in predicting outcomes in cancer patients treated with immunotherapy. Vaccines (Basel) 2020; 8: 203. 10.3390/vaccines8020203 PMC7349154 32353934

[35] Bajaj JS Heuman DM Hylemon PB, et al. The cirrhosis dysbiosis ratio defines changes in the gut microbiome associated with cirrhosis and its complications. J Hepatol 2014; 60: 940–947. 24374295 10.1016/j.jhep.2013.12.019 PMC3995845

[36] Chen Y Yang F Lu H, et al. Characterization of fecal microbial communities in patients with liver cirrhosis. Hepatology 2011; 54: 562–572. 21574172 10.1002/hep.24423

[37] Ponziani FR Bhoori S Castelli C, et al. Hepatocellular carcinoma is associated with gut microbiota profile and inflammation in nonalcoholic fatty liver disease. Hepatology 2019; 69: 107–120. 29665135 10.1002/hep.30036

[38] Fox JG Feng Y Theve EJ, et al. Gut microbes define liver cancer risk in mice exposed to chemical and viral transgenic hepatocarcinogens. Gut 2010; 59: 88–97. 19850960 10.1136/gut.2009.183749 PMC3891362

[39] Zheng Y Wang T Tu X, et al. Gut microbiome affects the response to anti-PD-1 immunotherapy in patients with hepatocellular carcinoma. J Immunother Cancer 2019; 7: 193. 31337439 10.1186/s40425-019-0650-9 PMC6651993

[40] Bellone M Calcinotto A Filipazzi P, et al. The acidity of the tumor microenvironment is a mechanism of immune escape that can be overcome by proton pump inhibitors. Oncoimmunology 2013; 2: e22058. 10.4161/onci.22058 PMC3583905 23483769

[41] Pilon-Thomas S Kodumudi KN El-Kenawi AE, et al. Neutralization of tumor acidity improves antitumor responses to immunotherapy. Cancer Res 2016; 76: 1381–1390. 26719539 10.1158/0008-5472.CAN-15-1743 PMC4829106

[42] Calcinotto A Filipazzi P Grioni M, et al. Modulation of microenvironment acidity reverses anergy in human and murine tumor-infiltrating T lymphocytes. Cancer Res 2012; 72: 2746–2756. 22593198 10.1158/0008-5472.CAN-11-1272

