# Supplementary material for: Antacid exposure and immunotherapy outcomes among patients with advanced hepatocellular carcinoma
Source: Ther Adv Med Oncol. 2021 Apr 28;13:17588359211010937. doi: 10.1177/17588359211010937 (PMC8107671; doi:10.1177/17588359211010937)
Supplement: sj-docx-1-tam-10.1177_17588359211010937 – Supplemental material for Antacid exposure and immunotherapy outcomes among patients with advanced hepatocellular carcinoma [file sj-docx-1-tam-10.1177_17588359211010937.docx]

Supplemental Table 1: Institutional Review Boards approving this study.

| Institutional Review Board | Location |
| --- | --- |
| Humanitas Research Hospital | Milan, Italy |
| Icahn School of Medicine at Mount Sinai | New York, USA |
| Imperial College London | London, UK |
| Taipei Veterans General Hospital | Taipei, Taiwan |
| University of Freiburg | Freiburg, Germany |
| University of Kansas Medical Center | Westwood, USA |
| Weill Cornell Medicine | New York, USA |
