# Supplementary material for: Antacid exposure and immunotherapy outcomes among patients with advanced hepatocellular carcinoma
Source: Ther Adv Med Oncol. 2021 Apr 28;13:17588359211010937. doi: 10.1177/17588359211010937 (PMC8107671; doi:10.1177/17588359211010937)
Supplement: sj-docx-2-tam-10.1177_17588359211010937 – Supplemental material for Antacid exposure and immunotherapy outcomes among patients with advanced hepatocellular carcinoma [file sj-docx-2-tam-10.1177_17588359211010937.docx]

Supplemental Table 2: Additional details on baseline antacid exposure for a subset of antacid-exposed patients in the cohort.

|  | N (%) |
| --- | --- |
| Baseline antacid exposure | 76 (100%) |
| USA | 26 (34.2%) |
| Europe | 33 (43.4%) |
| Asia | 17 (22.4%) |
| Baseline PPI only | 58 (76.3%) |
| Baseline H2RA only | 12 (15.8%) |
| Baseline PPI and H2RA exposure | 6 (7.9%) |
| Duration: <1 week | 5 (6.6%) |
| Duration: 1 to 4 weeks | 12 (15.8%) |
| Duration: >4 weeks | 59 (77.6%) |
| Indication: GI bleeding | 1 (1.3%) |
| Indication: Acid reflux/ dyspepsia | 33 (43.4%) |
| Indication: Procedure/ Prophylaxis | 37 (48.7%) |
| Indication: Other | 5 (6.6%) |
