# Supplementary material for: Antacid exposure and immunotherapy outcomes among patients with advanced hepatocellular carcinoma
Source: Ther Adv Med Oncol. 2021 Apr 28;13:17588359211010937. doi: 10.1177/17588359211010937 (PMC8107671; doi:10.1177/17588359211010937)
Supplement: sj-docx-3-tam-10.1177_17588359211010937 – Supplemental material for Antacid exposure and immunotherapy outcomes among patients with advanced hepatocellular carcinoma [file sj-docx-3-tam-10.1177_17588359211010937.docx]

Supplemental Table 3: Additional details on concurrent antacid exposure for a subset of antacid-exposed patients in the cohort.

|  | N (%) |
| --- | --- |
| Concurrent antacid exposure | 117 (100%) |
| USA | 54 (46.2%) |
| Europe | 36 (30.8%) |
| Asia | 22 (18.8%) |
| Concurrent PPI only | 72 (62.6%) |
| Concurrent H2RA only | 24 (20.9%) |
| Concurrent PPI and H2RA exposure | 19 (16.5%) |
| Timing: Started before ICI | 68 (63.6%) |
| Timing: <30d after ICI | 18 (16.8%) |
| Timing: 31-60d after ICI | 6 (5.6%) |
| Timing: 61-90d after ICI | 2 (1.9%) |
| Timing: >90d after ICI | 13 (12.1%) |
| Indication: GI bleeding | 5 (4.3%) |
| Indication: Acid reflux/ dyspepsia | 44 (37.9%) |
| Indication: Procedure/ Prophylaxis | 43 (37.1%) |
| Indication: Other | 24 (20.7%) |
